# Immunonutritional Biomarkers in Primary Sjögren’s Syndrome Disease Activity: CALLY Index and HALP Score

**DOI:** 10.5152/ArchRheumatol.2025.25012

**Published:** 2025-09-01

**Authors:** Ayşegül Özdoğan Bircan, Şerife Şeyda Zengin Acemoğlu, İpek Türk, Hüseyin Turgut Elbek Özer

**Affiliations:** Division of Rheumatology, Department of Internal Medicine, Çukurova University Faculty of Medicine, Adana, Türkiye

**Keywords:** CALLY index, disease activity, HALP score, immunonutritional biomarkers, primary Sjögren’s syndrome

## Abstract

**Background/Aims:**

Primary Sjögren’s syndrome (pSS) is an autoimmune disease that can affect several systems. The purpose of this study was to examine the connection between primary Sjögren’s disease activity and the CRP-albumin-lymphocyte index (CALLY) and the HALP (hemoglobin, albumin, lymphocyte, and platelet) score, 2 novel immunonutritional indicators that have not yet been applied to rheumatological disease activation.

**Materials and Methods:**

This cross-sectional study included 89 patients with pSS and 113 age- and sex-matched individuals. The relationship between haematological, inflammatory, immunonutritional biomarkers, and disease activation was investigated, as were the differences between the groups.

**Results:**

Eighty-nine patients (96.6% female, mean age 53.4 ± 15.0 years) and 113 control subjects (97.3% female, mean age 50.8 ± 14.9 years) were included in the study. The levels of C-reactive protein (CRP), erythrocyte sedimentation rate, red cell distribution width, neutrophil lymphocyte ratio, monocyte lymphocyte ratio, platelet lymphocyte ratio, and C-reactive protein albumin ratio were significantly higher in the group of patients with pSS compared to the control group (all; *P* < .001). Systemic immune-inflammation index and systemic inflammatory response index were significantly elevated in the patient cohort compared to the control group (*P* = .002 and *P* = .048, respectively). The CALLY index and HALP score were negatively correlated with EULAR Sjögren’s syndrome disease activity index (*P *< .001 for both).

**Conclusion:**

The CALLY index and HALP score represent a novel approach to assessing disease activity and prognosis in a number of conditions, including stroke, myocardial infarction, osteoarthritis, pulmonary thromboembolism, asthma, chronic obstructive pulmonary disease, and many types of cancer. They may also be useful as a guide to disease activity and monitoring in pSS.

**Main Points**

- New biomarkers of disease activity and prognosis are needed in primary Sjögren’s syndrome (pSS).
- The CRP-albumin-lymphocyte (CALLY) index and the HALP (hemoglobin, albumin, lymphocyte, and platelet) score hold promise in the prediction of the prognosis and activity of pSS.
- Immunonutritional markers HALP score and CALLY index negatively correlate with disease activation in pSS.

## Introduction

Primary Sjögren’s syndrome (pSS) is an idiopathic chronic autoimmune disorder characterized by keratoconjunctivitis sicca and xerostomia resulting from malfunction of the lacrimal and salivary glands.^1^ The pSS typically affects middle-aged women in their fourth to sixth decade of life. The ratio of female to male patients is approximately 9 : 1.^2,3^ Furthermore, it may manifest as non-visceral (skin, musculoskeletal involvement) or visceral (neurological, renal, haematological, pulmonary, gastrointestinal, and cardiovascular) extraglandular involvement.^4^ The European League Against Rheumatism (EULAR) Sjögren’s Syndrome task group has recently developed 2 disease activity indices: the EULAR Sjögren’s Syndrome Patient Reported Index (ESSPRI) for patient symptoms and the EULAR Sjögren’s Syndrome Disease Activity Index (ESSDAI) for systemic aspects.^5^

Monocytes, lymphocytes, and neutrophils are important players in inflammation. Acute phase reactants, including erythrocyte sedimentation rate (ESR) and C-reactive protein (CRP), are commonly utilized as indicators of systemic inflammation in rheumatological disorders.^6^ The ratios of these blood cells such as neutrophil lymphocyte ratio (NLR), monocyte lymphocyte ratio (MLR), and platelet lymphocyte ratio (PLR) are also used as biomarkers associated with acute and/or chronic inflammation.^7^ Although red cell distribution width (RDW), one of the hemogram parameters, is typically used in the etiology of anemia, many studies have found an association among this parameter and systemic inflammation, making an indicator for disease activity, including cancer, inflammatory disorders, and cardiovascular diseases.^8^ Mean platelet volume (MPV), which is a part of complete blood count (CBC) analysis that can be easily obtained in daily routine, has also contributed to disease activation and prognosis in many studies.^9^ Both the prediction of new biomarkers and the prediction of disease prognosis are crucial for managing diseases and determining treatment costs, and the quest for new biomarkers is ongoing. Therefore, the systemic immune-inflammation index (SII) and systemic inflammatory response index (SIRI) were developed using the components of the CBC requested at routine controls.^10^ The SII primarily indicates the interaction between thrombocytosis, neutrophil-driven inflammation, and lymphocyte-mediated immune regulation. The SIRI incorporates monocyte count as well, providing a more complete picture of innate immune activation. Both indexes are accessible and economical indicators of systemic inflammation and are clinically relevant in various conditions, including malignancies, cardiovascular disease, and autoimmune disorders. The ratio of C-reactive protein to albumin ratio (CAR) is another inflammatory biomarker.^11^

The HALP (hemoglobin, albumin, lymphocyte, and platelet) score and CRP-albumin-lymphocyte (CALLY) index are new immune nutrition biomarkers that have been used in many diseases such as malignancies, myocardial infarction, dyslipidemia, stroke etc. in recent years.^12,13^ The HALP score focuses on evaluating a patient’s nutritional and hematological status. Including anemia markers, albumin levels, and immune cell counts, this score provides information about the patient’s overall systemic condition. In contrast, the CALLY index emphasizes systemic inflammatory activity, as well as nutritional and immune parameters. This offers a more inflammation-focused assessment of the clinical state. Both indices can be obtained through routine laboratory tests and are valuable tools for evaluating disease severity and prognosis. This study aimed to compare NLR, PLR, MLR, RDW, MPV, CAR, SII, and SIRI indexes with the new immune nutritional markers, HALP score and CALLY index values, and to investigate their relationship to disease severity in clinical practice. The purpose of this study was to investigate the association between immunonutritional markers and various clinical findings, disease activity, and demographic characteristics in individuals with pSS.

## Methods

### Ethical and Informed Consent

This research was allowed by Çukurova University Human Research Ethics Committee (Approval no: 146/40 Approval date: July 26, 2024). All participants in the study were provided with sufficient information regarding the purpose and scope of the investigation, and their verbal and written consent was duly obtained.

### Patient Data

This cross-sectional case-control research was carried out from July 2024 to October 2024 and involved 89 pSS patients and 113 healthy participants matched by age and sex. The American College of Rheumatology/European Alliance of Associations for Rheumatology (ACR/EULAR) categorization criteria for pSS were used to diagnose pSS individuals.^14^ Parameters for exclusion include the existence of an autoimmune disease concurrently, using more than 5 mg of prednisolone and comparable steroids, haematological and solid organ malignancy, hematological disease (thalassemia and sickle cell anemia, etc.), acute hepatitis, and chronic liver disease. Patients with serious renal impairment (glomerular filtration rate (GFR) ≤ 30 mL/min), atherosclerotic cardiovascular disease, uncontrolled diabetes mellitus, active infection, breastfeeding, pregnancy, recent history of blood transfusion and iron deficiency anemia, vitamin B12, and folate deficiency are excluded.

Information was requested from the participants on age, gender, duration of illness, and the presence of keratoconjunctivitis sicca and xerostomia symptoms. Records of immunosuppressive (IS), disease modifying anti-rheumatic drugs (DMARDs), and combination therapies were also contained in the data set. The DMARD group comprised hydroxychloroquine, methotrexate, and leflunomide, whereas the IS group consisted of azathiopurine and mycophenolate mofetil. Comorbidity indices in both groups were calculated using the Charlson comorbidity index, which consists of 17 items, including connective tissue disease.^15^ The ESSDAI composite score, a recognized global indicator determined based on symptoms and laboratory markers, was used to assess the disease activity in each patient.^16^ The patients were classified into 3 categories according to the ESSDAI score: low (score <5), moderate (score 5-13), and high disease activity (score ≥ 14).

Laboratory markers included CBC, CRP, ESR, serum albumin, creatinine, rheumatoid factor, antinuclear antibody, anti-SSA, and anti-SSB antibodies.

### Systemic Inflammatory and Immunonutritional Biomarkers

The following formulae were employed in order to calculate the indices utilized in the course of the study.

NLR: Neutrophil (10^9^/L)/lymphocyte (10^9^/L)

MLR: Monocyte (10^9^/L)/lymphocyte (10^9^/L)

PLR: Platelet (10^9^/L)/lymphocyte (10^9^/L)

SII: Neutrophil (10^9^/L) × platelet (10^9^/L)/lymphocyte (10^9^/L)

SIRI: Neutrophil (10^9^/L) × monocyte (10^9^/L)/lymphocyte (10^9^/L)

CAR: CRP (mg/dL)/albumin (g/L)

CALLY index: Albumin (g/L) × lymphocyte (10^9^/L)/CRP (mg/dL) × 10^4^

HALP score: Hemoglobin (g/L) × albumin (g/L) × lymphocyte (10^9^/L)/platelet(10^9^/L)

### Statistical Analysis

The Statistical Package for the Social Sciences (SPSS) 25.0 software program (IBM SPSS Corp.; Armonk, NY, USA) was used to execute the statistical analysis of the data. Continuous variables are expressed as mean ± standard deviation for normally distributed data or as median (interquartile range) for non-normally distributed data. Categorical variables are summarized as frequencies and percentages. For evaluating categorical data, the chi-square test was utilized. In order to determine whether the parameters being investigated had a typical distribution, the Shapiro–Wilk test was used. The Mann–Whitney *U* test was utilized in cases where the data did not show the normal distribution. The area under the curve (AUC), a measure of the predictive ability of PLR, MLR, NLR, SII, SIRI, CAR, HALP, and CALLY values in differentiating between patient groups were computed using receiver operating characteristic (ROC) analysis. As a consequence of the ROC analysis, threshold values were established for each variable, and sensitivity and specificity values were calculated at these points. The Spearman’s rho correlation test was applied to identify the link between continuous measurements. For all tests, the limit for statistical significance was set at *P* < .05.

## Results

The study comprised 113 controls matched by age and sex (97.3% female; average age 50.8 ± 14.9 years) and 89 pSS patients (96.6% female; average age 53.4 ± 15.0 years). In age and gender, there was not a statistically significant distinction between the patient and control groups (*P* = .195 and *P* = .766, correspondingly). Demographic data of both groups and clinical data of PSS groups are summarized in Table 1.

The majority of patients (78.7%) exhibited evidence of articular involvement. The immunonutritional, inflammatory, and laboratory results for both groups are summarized in Table 2.

The CRP, ESR, RDW, NLR, MLR, PLR, and CAR levels were significantly higher and MPV was significantly lower in the patient group compared to the control group (all *P* < .001), perhaps indicating prolonged immune-mediated changes in megakaryocyte activity or decreased platelet formation. The SII and SIRI were substantially higher in the patient group compared to the control (*P* = .002 and *P* = .048, respectively). The CALLY index and HALP score were significantly lower in the pSS group compared to the control group (both *P* < .001) (Table 2).

**Figure 1.**
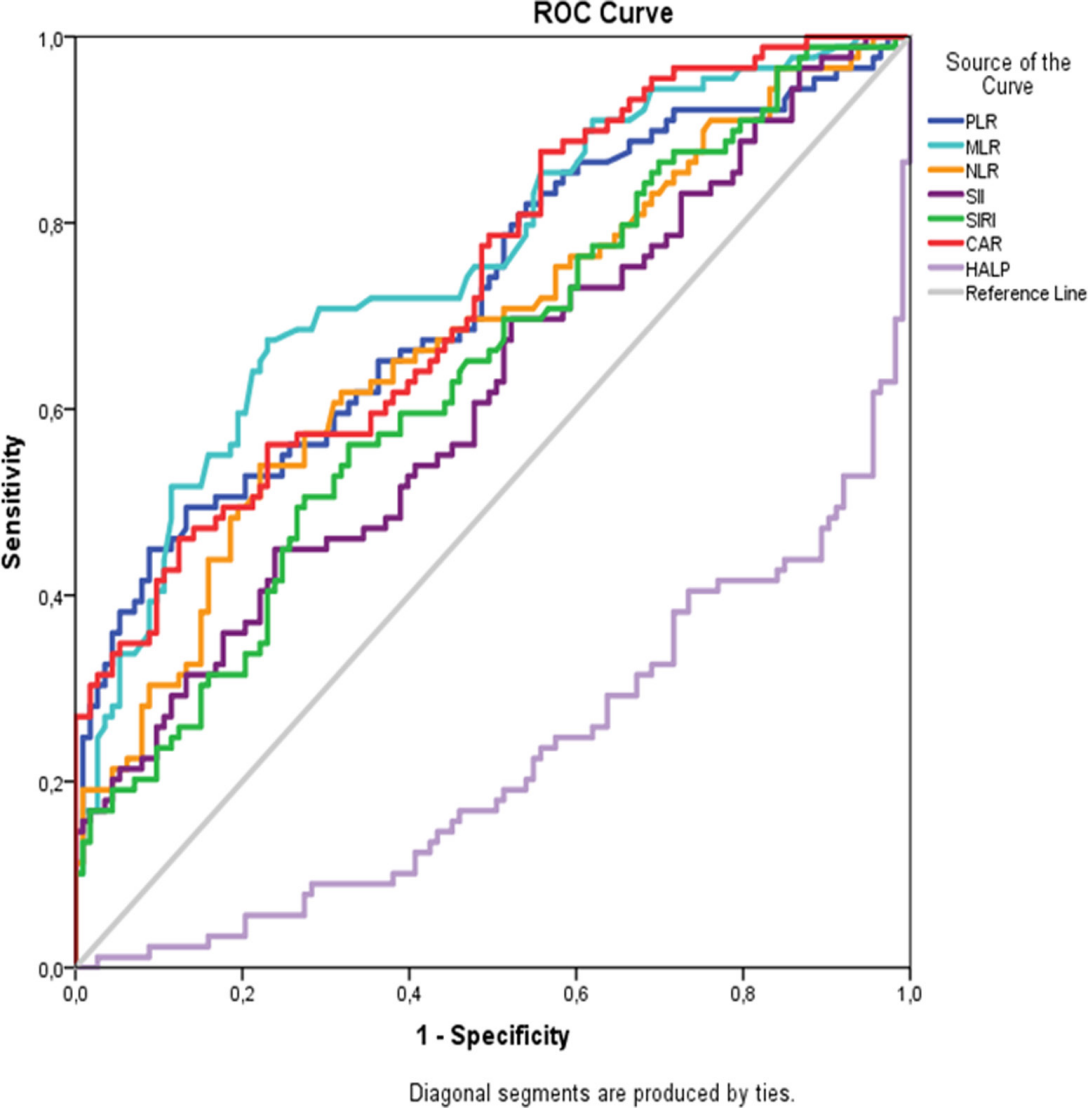
pSS patients ROC curve for inflammatory markers and HALP score.

**Figure 2.**
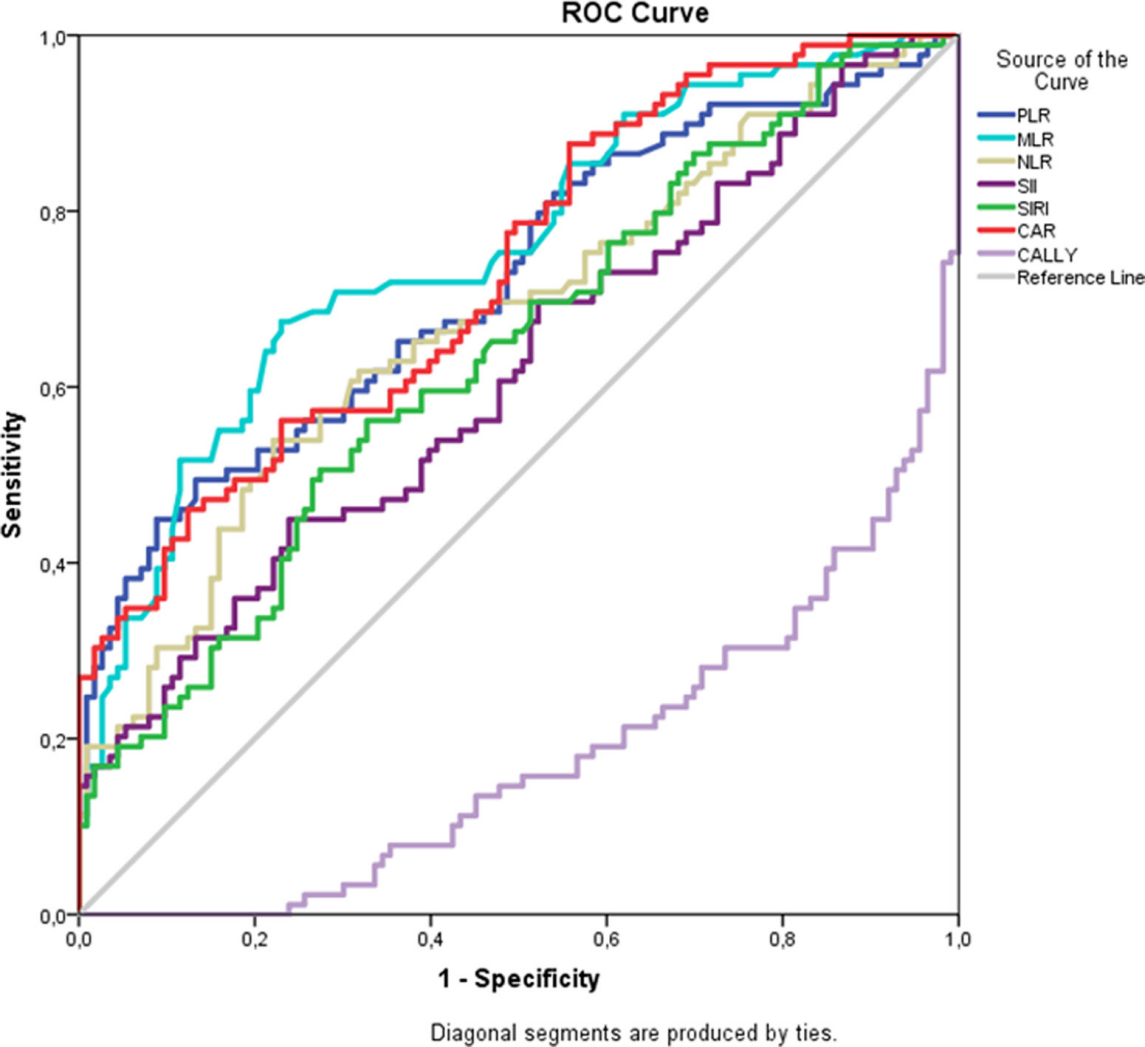
pSS patients ROC curve for inflammatory markers and CALLY index.

In order to evaluate the sensitivity, specificity, and accuracy of PLR, NLR, MLR, CAR, SII, SIRI, CALLY, and HALP in predicting disease activity in pSS patients, the optimal threshold value was determined; ROC curves were plotted and the AUC was used to assess the ability of the test to indicate disease severity (Figures 1 and 2).

The AUCs (95% CI) for PLR, NLR, and MLR were 0.719 (0.652-0.780), 0.676 (0.607-0.740), and 0.756 (0.690-0.813), respectively. The ROC curve analysis for SII resulted in an AUC of 0.615 (*P *= .004) with a sensitivity of 44.94% and a specificity of 76.11%, while for SIRI the AUC was 0.638 (*P *= .004) with a sensitivity of 56.18% and a specificity of 67.26%, and for CAR the AUC was 0.731 (*P* < .001) with a sensitivity of 46.07% and a specificity of 87.61%. The AUC was 0.775 (0.711-0.831) with a cut-off value of ≤33.5 for the HALP score. The CALLY index demonstrated an AUC of 0.819 (0.759-0.870), with a cut-off value of ≤0.21 (Table 3).

The PLR, MLR, NLR, SII, and SIRI had a positive correlation with ESSDAI disease activity, while HALP and the CALLY index were weakly negatively correlated with the same disease activity index (Table 4).

The CALLY index and the HALP score were evaluated separately along with the patients’ demographic data. A CALLY index ≤ 0.21 was observed in 62 patients (69.66%), with no statistically significant difference by gender (*P* = .245). There was no statistically substantial association between the CALLY index and patients’ treatments (*P* = .471). The age of patients with a CALLY index ≤ 0.21 was 56.7 ± 13.4 years, while CALLY > 0.21 was 45.9 ± 16.0 (*P* = .004). Disease duration and CALLY index were not considerably correlated (*P* = .704). In the group of patients with a CALLY index ≤ 0.21, the Charlson comorbidity index was higher than in those with a CALLY index > 0.21 (2.10 ± 1.2 vs. 1.48 ± 0.8; *P* = .020). A statistical comparison was made between CALLY and other inflammatory indices (Table 5).

Forty-nine of the 89 patients with pSS had a HALP score ≤ 33.5, and no statistically significant difference was found according to gender (*P* = .283). Regarding age, patients with a HALP score ≤ 33.5 were significantly older than those with a score > 33.5 (55.4 ± 15.5 vs. 50.5 ± 14.5 years, *P* = .021). There was no statistically significant relationship between HALP score and disease duration (*P* = .632). However, patients with a HALP score ≤ 33.5 had a higher mean Charlson comorbidity Index (1.82 ± 1.2) compared to those with a score > 33.5 (0.83 ± 1.0) (*P* < .001). The HALP score and CALLY index are also statistically compared with other inflammatory indices in Table 5.

## Discussion

Lymphocytic infiltration of exocrine glands is a hallmark of the complex autoimmune disease known as pSS.^17^ Predicting disease activity and evaluating treatment options are essential for the effective management of the disease. Several studies have demonstrated that CRP levels are elevated in inflammatory diseases and increase significantly in response to inflammation, injury, and infection.^18^ However, a study involving 53 patients with pSS and 47 healthy controls reported significantly higher levels of malondialdehyde and nitric oxide in the patient group, whereas CRP levels did not differ significantly between the groups.^19^ Therefore, identifying reliable biomarkers for disease activity continues to be an important area of research in rheumatology, particularly in pSS. This study investigated the association between disease activity and the CALLY index, the HALP score, and other hematological and immunological biomarkers that have demonstrated clinical relevance in the context of pSS, with the findings presented accordingly.

In the present study, the patient group had statistically significant higher levels of the NLR, PLR, and MLR than the control group. Consistent with the findings, Mihai et al^7^ evaluated 124 patients with pSS and 121 healthy controls and reported that the levels of NLR, PLR, and MLR were significantly increased in the pSS group, suggesting that these hematological ratios may serve as reliable indicators of systemic inflammation in pSS. The results were associated with low lymphocyte counts, reflecting in chronic inflammation, in the pSS patient group. Autophagy abnormalities, one of the factors contributing to the pathophysiology of autoimmune disease, could be the reason of the low lymphocyte count. Peripheral blood T lymphocyte autophagy levels may be linked to therapy responsiveness, lymphoma formation, and disease activity.^20^

The SII and the SIRI comprise major cellular elements of both innate and adaptive immunity. The SII includes neutrophils, lymphocytes, and platelets, serving as a composite marker that reflects the intensity of the inflammatory response, as indicated by neutrophil and platelet levels, and the immune regulatory function associated with lymphocytes. The SIRI, on the other hand, is calculated using neutrophil, monocyte, and lymphocyte counts, allowing for the assessment of inflammation along with the contribution of monocytes to innate immune responses. These indices offer additional insight into the relationship between thrombocytosis, inflammation, and immune dysregulation observed in autoimmune diseases.^21^ In Mercader-Salvans et al’s^22^ research, involving 284 systemic lupus erythematosus patients and 181 controls matched by age and sex, the patient group’s NLR, MLR, and PLR were found to be statistically considerably higher, while the patient group’s SIRI was higher but not significantly different. A total of 116 participants in a study that included people with rheumatoid arthritis (RA) in remission, active RA patients, and a control group found that the active patients had a statistically important SII higher compared to the remission group, and all patients had a higher SII versus the controls.^23^ It’s currently unknown how SII and SIRI predict disease activity and prognosis for people with pSS. However, various studies have shown its connection to the prognosis of autoimmune disorders. According to the research, SII and SIRI showed a positive connection with ESSDAI and were considerably higher in the pSS patient group than in the controls.

Iida et al^24^ initially investigated the CALLY index for prognostication prior to hepatectomy in hepatocellular cancer in order to evaluate nutritional status, inflammation, and immunology. The CALLY index has been found to be statistically significant and negatively associated with disease activation, stage, and prognosis in studies of myocardial infarction and various cancers.^25-27^ This research is the initial and only one that uses the CALLY index in pSS, as far as we are aware. The findings of the study may be explained by several contributing factors, including malnutrition associated with burnout syndrome, dry mouth, impaired taste and swallowing functions,^28^ chronic inflammation, and lymphocyte autophagy.

Another simple but effective immunonutritional marker of the body’s basic nutritional and inflammatory state is the HALP score. Around 13,110 patients from 28 trials (31 cohorts) were included in the meta-analysis, which found that the pretreatment HALP score was a significant and unfavorable predictive indicator for decreased cancer survival.^29^ In a retrospective study of 212 IS drug-naive patients with antineutrophil cytoplasmic antibody-associated vasculitis, the HALP score was thought to better show the level of inflammation and dynamic changes in disease activity during laboratory testing.^30^

This study has many strengths. To the best of knowledge, this is the first study to examine the potential utility of HALP score and CALLY index as immunonutritional markers in patients with pSS. The use of routine tests based on various hematological and inflammatory indices facilitates interpretation and enhances their potential relevance in clinical practice.

However, the study also has limitations. Being cross-sectional, the study cannot determine if the observed relationships are causal. In addition, its single-center design and the relatively small number of patients, especially males, may limit the generalizability of the findings. The results may also have been influenced by unmeasured factors, particularly nutritional status.

In conclusion, the study revealed that HALP score and CALLY index showed statistically significant lower scores in the pSS group compared to the controls and were inversely correlated with disease activity. These findings suggest that HALP score and CALLY index, as accessible immunonutritional indices, may offer additional insight into systemic inflammation and disease activity in pSS. However, as they are based on non-specific laboratory parameters, they may not fully reflect the complex immune processes involved in the disease. Incorporating more specific biomarkers into these indices could enhance their diagnostic and prognostic utility. Future multicenter studies with larger and more diverse patient cohorts are needed to validate these results and further explore their clinical applicability.

## Figures and Tables

**Table 1.** Demographic and Clinical Data of pSS and Control Groups

Demographic Data	pSS (n = 89)	Control Group (n = 113)	*P*
Age (years), mean ± SD	53.4 ± 15.0	50.8 ± 14.9	.195
Female sex, n (%)	86 (96.6)	110 (97.3)	.766
Comorbidity index, median (min-max)	1 (1-5)	0 (0-3)	<.001**
Dry mouth, n (%)	56 (62.9)		
Dry eyes, n (%)	70 (78.7)		
Treatment, n (%) Untreated DMARDs IS DMARDs + IS	1 (1.1) 64 (71.9) 13 (14.6) 11 (12.4)		
Disease duration (month), median (min-max)	39 (0-165)		
ESDAII score, Median (min-max) <5, n (%) 5-13, n (%) ≥14, n (%)	4 (0-23) 47 (52.8) 31 (34.8) 11 (12.4)		

DMARDs, disease modifying anti-rheumatic drugs; ESSDAI, EULAR Sjögren's syndrome disease activity index; IS, immunosuppressive.

*P. < .05.

**P < .01, Mann–Whitney *U*.

**Table 2.** pSS Patient and Control Group Laboratory Features, Inflammatory, and Immunonutritional Indices

	pSS (n = 89)	Control Group (n = 113)	*P*
ANA Positive Negative	76 (85.4) 13 (14.6)		
anti-SSA Positive Negative	64 (71.9) 25 (28.1)		
anti-SSB Positive Negative	26 (29.2) 63 (70.8)		
RF Positive Negative	25 (28.1) 64 (71.9)		
Laboratory features WBC (10^3^/µL) Lymphocyte (10^3^/µL) Monocyte (10^3^/µL) Neutrophil (10^3^/µL) Basophil (10^3^/µL) Eosinophil (10^3^/µL) Platelets (10^3^/µL) Hemoglobin (g/dL) RDW (%) MPV (fL) Albumin (g/L) Creatinin (mg/dL) CRP (mg/L) ESR (mm/hour)	6.09 ± 1.6 1.60 ± 0.6 0.52 ± 0.2 3.73 ± 1.3 0.0 (0.0-0.0) 0.1 (0-6) 263.6 ± 74.5 12.5 ± 1.12 14.9 ± 1.6 8.90 ± 0.9 39.5 ± 4.8 0.64 ± 0.18 4.2 (1-30.8) 18 (2-87)	6.95 ± 1.7 2.28 ± 0.6 0.51 ± 0.16 3.95 ± 1.3 0.03 (0-1) 0.12 (0.0-0.9) 274.8 ± 70.5 12.9 ± 1.14 14.1 ± 1.6 9.74 ± 1.3 43.7 ± 3.2 0.64 ± 0.14 2.53 (0.2-11.6) 12 (2-37)	<.001** <.001** .818 .155 <.001** .080 .233 .004* <.001** <.001** <.001** .632 <.001** <.001**
Ratios of cells NLR MLR PLR	2.21 (1-14) 0.32 (0-3) 155.9 (65-726)	1.63 (1-5) 0.23 (0-1) 122.9 (49-292)	<.001** <.001** <.001**
Inflammatory indexes SII SIRI CAR	532 (224-5876) 1.07 (0-11) 1.01 (0-10)	447.3 (160-1203) 0.84 (0-3) 0.6 (0-2)	.005** .001** <.001**
Immunonutritional index CALLY HALP	0.15 (0-1) 32.6 ± 15.6	0.36 (0-5) 49.2 ± 17.0	<.001** <.001**

Values are presented in: n (%), mean ± standard deviation, or median (min-max)

ANA, antinuclear antibodies; anti-SSA, anti-SSA antibodies; anti-SSB, anti-SSB antibodies; CAR, C-reactive protein/albumin ratio; CALLY, CRP-albumin-lymphocyte; CRP, C-reactive protein; ESR, erythrocyte sedimentation rate; HALP, hemoglobin-albumin-lymphocyte-platelet; MLR, monocyte lymphocyte ratio; MPV, mean platelet volume; NLR, neutrophil lymphocyte ratio; PLR, platelet lymphocyte ratio; RDW, red cell distribution width; RF, rheumatoid factor; SII, systemic immune-inflammation index; SIRI, systemic inflammatory response index; WBC, white blood cell.

*P < .05.

**P < .01. Mann–Whitney *U*.

**Table 3.** Differences Between pSS Patients and Controls Using Inflammatory and Immunological Markers

	Cutoff	AUC (95% CI)	Sensitivity (95% CI)	Specificity (95% CI)	PPV (95% CI)	NPV (95% CI)	*P*
PLR	>161.9	0.719 (0.652-0.780)	49.44 (38.7-60.2)	86.73 (79.1-92.4)	74.6 (63.6-83.1)	68.5 (63.7-73.0)	<.001*
MLR	>0.273	0.756 (0.690-0.813)	67.42 (56.7-77)	76.99 (68.1-84.4)	69.8 (61.5-76.9)	75 (68.6-80.4)	<.001*
NLR	>2.13	0.676 (0.607-0.740)	53.93 (43-64.6)	77.88 (69.1-85.1)	65.8 (56.4-74)	68.2 (62.7-73.3)	<.001*
SII	>613.03	0.615 (0.544-0.682)	44.94 (34.4-55.9)	76.11 (67.2-83.6)	59.7 (49.8-68.9)	63.7 (58.6-68.5)	.004*
SIRI	>0.997	0.638 (0.568-0.704)	56.18 (45.3-66.7)	67.26 (57.8-75.8)	57.5 (49.5-65.1)	66.1 (59.8-71.8)	.004*
CAR	>1.19	0.731 (0.665-0.791)	46.07 (35.4-57)	87.61 (80.1-93.1)	74.5 (63.1-83.4)	67.3 (62.7-71.4)	<.001*
HALP	<33.5	0.775 (0.711-0.831)	56.18 (45.3-66.7)	89.38 (82.2-94.4)	80.6 (70.3-88)	72.1 (67-76.8)	<.001*
CALLY	<0.21	0.819 (0.759-0.870)	69.66 (59-79)	80.53 (72-87.4)	73.8 (65.4-80.8)	77.1 (70.8-82.4)	<.001*

CALLY, CRP-albumin-lymphocyte; CAR, C-reactive protein/albumin ratio; HALP, hemoglobin-albumin-lymphocyte-platelet; MLR, monocyte lymphocyte ratio; NLR, neutrophil lymphocyte ratio; NPV, negative predictive value; PLR, platelet lymphocyte ratio; PPV, positive predictive value; SII, systemic immune-inflammation index; SIRI, systemic inflammatory response index.

*P < .05, ROC curve.

**Table 4.** Correlation of Inflammatory and Immunonutritional Indices with ESSDAI Score

	*r*	*P*
PLR	0.244	.021*
MLR	0.239	.024*
NLR	0.252	.017*
SII	0.308	.003**
SIRI	0.260	.014*
CAR	0.463	<.001**
HALP	−0.380	<.001**
CALLY	−0.473	<.001**

CALLY, CRP-albumin-lymphocyte; CAR, C-reactive protein/albumin ratio; HALP, hemoglobin-albumin-lymphocyte-platelet; MLR, monocyte lymphocyte ratio; NLR, neutrophil lymphocyte ratio; PLR, platelet lymphocyte ratio; r, correlation coefficient; SII, systemic immune-inflammation index; SIRI, systemic inflammatory response index.

*P. < .05

**P < .01. Spearman’s rho.

**Table 5.** Relationships Between CALLY Index and HALP Score and Other Markers of Inflammation

	CALLY ≤ 0.21 (n = 62) Mean ± SD	CALLY > 0.21 (n = 27) Mean ± SD	*P*	HALP ≤ 33.5 (n = 49) Mean ± SD	HALP > 33.5 (n = 40) Mean ± SD	*P*
PLR	229.6 ± 149.6	130.1 ± 35.3	<.001**	248.5 ± 140.5	119.3 ± 28.4	<.001**
MLR	0.42 ± 0.35	0.29 ± 0.09	.015*	0.43 ± 0.35	0.24 ± 0.08	<.001**
NLR	3.31 ± 2.7	1.94 ± 0.70	.004**	3.50 ± 2.7	1.78 ± 0.66	<.001**
SII	951.8 ± 1034.4	469.9 ± 209.6	.002**	1045.8 ± 1006.6	455.9 ± 198.0	<.001**
SIRI	1.72 ± 1.8	1.04 ± 0.6	.048*	1.80 ± 1.8	0.94 ± 0.50	<.001**
CAR	2.32 ± 2.0	0.57 ± 0.23	<.001**	2.12 ± 2.1	0.79 ± 0.54	<.001**

CALLY, CRP-albumin-lymphocyte; CAR, C-reactive protein/albumin ratio; HALP, hemoglobin-albumin-lymphocyte-platelet; MLR, monocyte lymphocyte ratio; NLR, neutrophil lymphocyte ratio; PLR, platelet lymphocyte ratio; SII, systemic immune-inflammation index; SIRI, systemic inflammatory response index.

*P < .05.

**P < .01. Mann–Whitney *U.*

## Data Availability

The datasets gathered during the preparation of this manuscript are available from the corresponding author upon reasonable request.
